# Genetic variability of *HIF1A* and response to treatment with cisplatin in combination with pemetrexed or gemcitabine in patients with malignant mesothelioma

**DOI:** 10.2478/raon-2025-0049

**Published:** 2025-09-05

**Authors:** Matic Setina, Eva Setina, Ziga Doljak, Katja Goricar, Vita Dolzan, Viljem Kovac

**Affiliations:** Faculty of Medicine, University of Ljubljana, Ljubljana, Slovenia; Pharmacogenetics Laboratory, Institute of Biochemistry, Faculty of Medicine, University of Ljubljana, Ljubljana, Slovenia; Institute of Oncology Ljubljana, Ljubljana, Slovenia

**Keywords:** malignant mesothelioma, chemotherapy, hypoxia-inducible factor, polymorphism

## Abstract

**Background:**

Treatment of malignant mesothelioma (MM) still relies on chemotherapy with cisplatin in combination with pemetrexed or other drugs. Studies indicate that hypoxic conditions within tumour tissue may reduce responsiveness to cisplatin-based chemotherapy. Hypoxia-inducible factors (HIF) play an important role in regulation of cellular adaptation to hypoxia. The aim of our study was to investigate single nucleotide polymorphisms (SNPs) in the *HIF1A* gene coding for the regulatory alpha subunit (HIF-1A) and their role in the response to chemotherapy in patients with MM.

**Patients and methods:**

Our retrospective genetic association study included 234 patients with MM, who were treated with a combination of cisplatin/pemetrexed or cisplatin/gemcitabine at the Institute of Oncology Ljubljana between January 2001 and September 2018. Selected *HIF1A* SNPs (rs1154965, rs11549467, and rs2057482) were genotyped using the competitive allele-specific polymerase chain reaction (KASP). Additionally, we used a TaqMan assay for independent confirmation of rs11549465 genotyping results. The impact of the SNPs on response to chemotherapy was analysed using logistic regression. For survival analysis, we used the Kaplan-Meier method and Cox regression.

**Results:**

In heterozygotes with the *HIF1A* rs11549465 CT genotype, response to chemotherapy was significantly worse compared to homozygotes with the CC genotype, but only after adjustment for weight loss and CRP (RO_adj_ = 0.37; 95% CI = 0.14–0.97; P_adj_ = 0.044). *HIF1A* rs11549467 and rs2057482 were not associated with response to chemotherapy (all P > 0.05). None of the investigated SNPs were associated with progression-free survival or overall survival (all P > 0.05).

**Conclusions:**

Among the investigated *HIF1A* SNPs, only rs11549465 has showed association with a worse response to chemotherapy after the adjustment for clinical parameters. The findings of this study have improved our understanding of the role of *HIF1A* polymorphisms in MM and may offer valuable insights into their impact on other cancers as well.

## Introduction

Malignant mesothelioma (MM) is a rare and highly aggressive cancer that most commonly arises from the mesothelial cells of the pleura.^1,2^ In vast majority of cases, it is associated with direct occupational or environmental exposure to asbestos.^3,4^ MM has a long latency period, and its incidence continues to rise both in Slovenia and worldwide.^3,5,6^ It typically presents with dyspnoea, chest pain, weight loss, and fatigue. Most patients are already in an advanced stage of the disease at the time these symptoms appear.^7,8^

The cornerstone of treatment for MM remains systemic chemotherapy with cisplatin, mostly combined either with pemetrexed or gemcitabine. Chemotherapy is part of a multimodal treatment approach for patients with operable MM, administered either before or after surgery. Combination chemotherapy has proven to be more effective than monotherapy. Additionally, chemotherapy is used for palliative treatment in patients with inoperable disease, advanced stages, or those who decline surgery. In the 1990s, a trimodal treatment approach became established for operable MM, which includes adjuvant or neoadjuvant chemotherapy, surgery, and adjuvant radiotherapy.^9–12^ Systemic treatment combining chemotherapy and immune checkpoint inhibitors (ICIs) is often chosen for patients with inoperable or recurrent MM who are in good general condition. Early inclusion of patients receiving systemic treatment for inoperable MM in palliative care is important, as it has been shown to prolong survival.^11,13–15^ Additionally, a newer treatment option for inoperable MM is immunotherapy using ICIs such as nivolumab plus ipilimumab, which can be used as first-line therapy or introduced in the second line of treatment.^12,16–18^

Despite advances in chemotherapy and immunotherapy, MM remains largely incurable, prompting ongoing research into biological markers for more personalized treatment.^12,19,20^ Hypoxia was shown to reduce the responsiveness of MM cells to cisplatin while increasing their invasiveness and the synthesis of hypoxia-inducible factor 1 (HIF-1), suggesting that HIF-1 could represent a significant target for potential new therapeutic approaches in MM.^21^

The predominance of tumor cell proliferation over angiogenesis, along with the abnormal structure and distribution of the capillary network in MM tissue, creates hypoxic conditions to which tumor cells adapt in various ways. The primary homeostatic response to hypoxia is mediated by transcription factors from the hypoxia-inducible factor (HIF) family. These intracellular proteins become activated under hypoxic conditions, bind to DNA, and regulate gene expression, enabling cells to adapt to oxygen deprivation. HIFs exist in three different isoforms, with HIF-1 expressed in all cells, while HIF-2 and HIF-3 are present only in certain tissues. Expression of HIF-1 in tumors increases the transcription of genes encoding enzymes, growth factors, and other proteins, significantly influencing tumor growth, survival, angiogenesis, and metastasis.^22–24^

HIF-1 is a heterodimeric protein composed of an oxygen-regulated alpha subunit (HIF-1A) and an oxygen-insensitive, constitutively expressed beta subunit (HIF-1B). It is tightly regulated to ensure its effects occur only under hypoxic conditions.^22,25^ Genetic variants may impair HIF-1 regulation, resulting in HIF-1 being active even under normoxic conditions, leading to autonomous heterodimerization of the HIF-1 subunits, DNA binding, and transcription of target genes independent of oxygen levels in the cells.^23^

Genetic variants, most commonly single nucleotide polymorphisms (SNPs) in the gene encoding the alpha subunit of HIF-1 (*HIF1A*), were reported to be associated with various diseases, with notable links to different malignancies. The most frequently studied SNPs are rs11549465 and rs11549467. In SNP rs11549465, cytosine (C) at position 1772 is replaced by thymine (T), resulting in the substitution of proline 582 with serine (p.Pro582Ser) within the oxygen-dependent degradation domain (ODD). In SNP rs11549467, adenine (A) at position 1790 is replaced by guanine (G), leading to the replacement of alanine 588 with threonine (p.Ala588Thr) in the ODD.^23^ Another SNP often linked to cancer is rs2057482 (c.A395G), which involves an A to G substitution in the 3’ untranslated region (3’ UTR) of the gene.^26^

A review study that analyzed 97 association studies identified links between 16 *HIF1A* SNPs and 40 disease phenotypes, including 14 types of cancer, such as breast cancer, lung cancer, colorectal cancer, stomach cancer, prostate cancer, oral cavity cancer, and renal cell carcinoma. Among the most frequently reported cancer-associated SNPs are *HIF1A* rs11549465, rs11549467, and rs2057482.^23^ Additionally, studies have found that *HIF1A* SNPs are associated with worse disease prognosis of cancer patients and an increased risk of metastasis.^23,27,28^ Most studies conducted to date have focused on the association of HIF-1A with cancer risk, but only a few investigated treatment response and survival. To date, however, no study has examined the role of HIF-1A in MM.

The aim of this study was to investigate the role of HIF-1A in the response to chemotherapy in patients with MM. We hypothesized that *HIF1A* polymorphisms are associated with the response to treatment with a combination of cisplatin and pemetrexed or gemcitabine in patients with MM.

## Patients and methods

We conducted a retrospective genetic association study. Our study population consisted of 234 patients with MM who received treatment with cisplatin combined with either pemetrexed or gemcitabine between January 1st, 2001, and September 30, 2018, at the Institute of Oncology Ljubljana, Slovenia. Diagnostic tissue samples from patients with pleural MM were collected using medical thoracoscopy or video-assisted thoracoscopic surgery (VATS), while samples for peritoneal MM were obtained using laparoscopic or open surgical procedures. Confirmation of diagnosis was performed through histopathological evaluation supported by immunohistochemical staining. Comprehensive clinical data, including patient demographics, therapeutic regimens, treatment responses, survival outcomes and adverse events, were extracted from patients’ medical records of the Institute of Oncology Ljubljana and the Cancer Registry of the Republic of Slovenia. Treatment response was categorized into complete response (CR), partial response (PR), stable disease (SD), and progressive disease (PD).

### Molecular genetic analysis

Genomic DNA from patients with MM included in this study had been isolated from the patients’ peripheral venous blood following standard extraction protocols during previous studies.^29^ Based on a review of relevant literature using the PubMed database, three common *HIF1A* SNPs were selected for genotyping: rs11549465 (p.Pro582Ser; C1772T), rs11549467 (p.Ala588Thr; A1790G), and rs2057482 (c.A395G). Allele and genotype frequencies in the European population were obtained from the NCBI dbSNP database. Genotyping of the selected SNPs was performed using the competitive allele-specific polymerase chain reaction (KASP) SNP Genotyping Assays (LGC Group). To validate the results obtained by KASP, rs11549465 was additionally genotyped using the 5’-exonuclease (TaqMan) Genotyping Assay (LGC Group) as an independent confirmation method.

### Statistical analysis

Continuous variables were described using median values and interquartile ranges, while categorical variables were described using frequencies. Minor allele frequency (MAF) was calculated for each SNP and conformity with Hardy-Weinberg equilibrium (HWE) was assessed using the chisquare (χ^2^) test. Statistical analyses were conducted using both additive and dominant models. The influence of SNPs on chemotherapy response was evaluated using logistic regression. Univariable logistic regression was used to assess the independent effect of each SNP, with results expressed as odds ratios (OR) and corresponding 95% confidence intervals (CI). In cases where any subgroup contained fewer than five individuals, Fisher’s exact test was applied instead of logistic regression. Multivariable logistic regression was performed to adjust for clinical factors that demonstrated a statistically significant association with treatment response. Covariates included in the multivariable models were selected using a stepwise forward conditional selection method. Survival analysis was performed using Kaplan–Meier estimation and Cox proportional hazards regression. Median survival time and follow-up duration were calculated using the Kaplan–Meier method. Hazard ratios (HR) and 95% confidence intervals were estimated using Cox regression analysis. All tests were two-tailed. P-values less than 0.05 were considered as statistically significant. Statistical analyses were conducted using IBM SPSS Statistics version 27.0 (IBM Corporation, Armonk, NY, USA).

## Results

### Patients

Our study included 234 patients with MM who were treated with a combination of either cisplatin and pemetrexed or cisplatin and gemcitabine. Their characteristics are summarized in Table 1.

**TABLE 1.** Clinical characteristics of patients with malignant mesothelioma (N = 234)

Characteristic	Category/Unit	N (%) [N]
**Sex**	Man, N (%)	176 (75.2)
	Woman, N (%)	58 (24.8)
**Age**	Age, median (25%– 75%)	66 (58–72)
**Stage**	I	15 (6.4) [1]
	II	53 (22.7)
	III	75 (32.2)
	IV	62 (26.6)
	Peritoneal MM	28 (12.0)
**Histological type**	Epithelioid	183 (78.2)
	Biphasic	20 (8.5)
	Sarcomatoid	22 (9.4)
	Not specified	9 (3.8)
**ECOG performance status**	0	15 (6.4)
	1	126 (53.8)
	2	90 (38.5)
	3	3 (1.3)
**C-reactive protein (CRP)**	Median (25%–75%)	21 (8–59) [19]
**Asbestos exposure**	No, N (%)	60 (25.8) [1]
	Yes, N (%)	173 (74.2)
**Smoking**	No, N (%)	129 (55.6) [2]
	Yes, N (%)	103 (44.4)
**Pain**	No, N (%)	99 (43.4) [6]
	Yes, N (%)	129 (56.6)
**Weight loss**	No, N (%)	76 (34.4) [13]
	Yes, N (%)	145 (65.6)
**Type of chemotherapy**	Gemcitabine/cisplatin	152 (65.0)
	Pemetreksed/cisplatin	82 (35.0)
**Response to chemotherapy**	SD+PD	147 (65.0) [8]
	CR+PR	79 (35.0)
**Disease progression**	No, N (%)	21 (9.1) [3]
	Yes, N (%)	210 (90.9)
**Death**	No, N (%)	65 (27.8)
	Yes, N (%)	169 (72.2)
**Progression-free survival**	Months, median value (25%–75%)	7.9 (5.3–13.8)
**Overall survival**	Months, median value (25%–75%)	18.2 (9.7–28.0)
**Follow-up duration**	Months, median valu (25%–75%)	44.4 (20.4–75.5)

1 CR = complete response; ECOG = Eastern Cooperative Oncology Group; N = number of patients; [N] = number of patients with missing data for the given parameter; PD = progressive disease; PR = partial response; SD = stable disease

Among the included patients, 176 (75.2%) were male and 58 (24.8%) were female, and their median age was 66 years. Only 15 patients (6.4%) were diagnosed at stage I, while the majority were diagnosed at more advanced stages of the disease. Notably, 28 patients (12.0%) had peritoneal MM. Among the histological subtypes, the epithelioid type was predominant, confirmed in 183 patients (78.2%), followed by the sarcomatoid type in 22 patients (9.4%) and the biphasic type in 20 patients (8.5%). In 9 patients (3.8%), the histological subtype could not be determined. Asbestos exposure was confirmed in 173 patients (74.2%), and 103 patients (44.4%) were smokers. Based on the Eastern Cooperative Oncology Group (ECOG) performance status, most patients fell into category 1 (126 patients, 53.8%) or category 2 (90 patients, 38.5%). The median C-reactive protein (CRP) level was 21 mg/L. The majority of patients (65.0%) were treated with a combination of cisplatin and gemcitabine, while the remaining 35.0% received cisplatin and pemetrexed. A complete or partial response to chemotherapy was achieved in 79 patients (35.0%), while 147 patients (65.0%) had stable disease or disease progression despite treatment. The median progression-free survival was 7.9 months, and the median overall survival was 18.2 months.

### Genotype frequency distribution

The genotype frequency distribution of the three investigated *HIF1A* SNPs is presented in Supplementary Table 1. Genotyping was not successful for rs11549465 in 22 patients, for rs11549467 in 13 patients, and for rs2057482 in 5 patients. The genotype distributions for rs11549467 and rs2057482 conformed to HWE. In contrast, the rs11549465 genotype distribution deviated from HWE and was not representative of European population, as there were more homozygotes for the polymorphic allele (TT) in our group.

### Association of the investigated *HIF1A* SNPs with response to chemotherapy

We observed a statistically significantly poorer response to chemotherapy in patients carrying the rs11549465 CT genotype compared to those carrying the more common CC genotype, but only after adjustment for weight loss and CRP levels (OR_adj_ = 0.37; 95% CI]: 0.14-0.97; P_adj_ = 0.044). Among rs11549465 CC genotype carriers, 37.8% achieved a complete or partial response, but this proportion was only 22.5% among rs11549465 CT genotype carriers. For the other polymorphisms, no statistically significant associations with chemotherapy response were found (Table 2).

**TABLE 2.** Association between investigated single nucleotide polymorphisms with response and chemotherapy (N = 226)

SNP	Genotype	SD/PD N (%)	CR/PR N (%)	OR (95% CI)	P	OR_adj_ (95% CI)_adj_	P_adj_
**rs11549465**	CC	92 (62.2)	56 (37.8)	Ref.		Ref.	
	CT	31 (77.5)	9 (22.5)	0.48 (0.21–1.08)	0.074	0.37 (0.14–0.97)	**0.044**
	TT	10 (62.5)	6 (37.5)	0.99 (0.34–2.86)	0.979	1.55 (0.48–4.98)	0.464
	CT+TT	41 (73.2)	15 (26.8)	0.60 (0.31–1.18)	0.141	0.60 (0.28–1.30)	0.194
**rs11549467**	GG	126 (65.6)	66 (34.4)	Ref.		Ref.	
	GA+AA	14 (66.7)	7 (33.3)	0.96 (0.37–2.48)	0.924	1.06 (0.38–2.98)	0.906
**rs2057482**	CC	95 (63.3)	55 (36.7)	Ref.		Ref.	
	CT	41 (66.1)	21 (33.9)	0.89 (0.48–1.65)	0.699	0.92 (0.45–1.85)	0.806
	TT	7 (70.0)	3 (30.0)	0.74 (0.18–2.98)	0.672	0.61 (0.11–3.39)	0.574
	CT+TT	48 (66.7)	24 (33.3)	0.86 (0.48–1.56)	0.627	0.87 (0.45–1.71)	0.689

1 adj = adjusted for weight loss and CRP; CR = complete response; CI = confidence interval; N = number of patients; OR = odds ratio; PD = progressive disease; PR = partial response; Ref. = reference genotype; SD = stable disease; SNP = single nucleotide polymorphism

When we further analyzed the association between the *HIF1A* SNPs and response to treatment separately for patients treated with the combination of gemcitabine/cisplatin, our study showed no statistically significant associations, neither in the univariable analysis nor after the adjustment for weight loss and ECOG performance status in a multivariable regression analysis (Supplementary Table 2).

Similarly, when we separately analyzed the association between the *HIF1A* SNPs and response to treatment only for patients treated with the combination of pemetrexed/cisplatin, our study showed no statistically significant associations, neither in the univariable analysis, nor after the adjustment for pain in the multivariable regression analysis (Supplementary Table 3).

### Association of the investigated *HIF1A* SNPs with progression-free survival

No statistically significant association between polymorphisms and PFS was observed in our study, neither in the univariable analysis, nor after adjusting for CRP levels, histological subtype, weight loss, asbestos exposure, and smoking status in the multivariable analysis (Table 3), and nor when we separately analyzed patients treated with the combination of gemcitabine/cisplatin (Supplementary Table 4) and patients treated with the combination of pemetrexed/cisplatin (Supplementary Table 5).

**TABLE 3.** Association between investigated polymorphisms and progression-free survival (N = 231)

SNP	Genotype	PFS median (25–75%)	HR (95% CI)	P	HR_adj_ (95% Cl)_adj_	P_adj_
**rs11549465**	CC	7.8 (5.6–13.0)	Ref.		Ref.	
	CT	8.9 (5.3–16.2)	0.92 (0.64–1.33)	0.665	0.79 (0.52–1.20)	0.271
	TT	5.6 (3.5–17.4)	1.1 (0.69–1.90)	0.608	0.98 (0.55–1.72)	0.932
	CT+TT	8.5 (4.8–16.2)	0.98 (0.71–1.35)	0.912	0.84 (0.59–1.21)	0.354
**rs11549467**	GG	7.7 (5.2–13.1)	Ref.		Ref.	
	GA+AA	11.2 (7.7–16.0)	0.74 (0.46–1.19)	0.220	0.67 (0.40–1.11)	0.119
**rs2057482**	CC	8.9 (5.5–16.0)	Ref.		Ref.	
	CT	7.9 (5.3–12.4)	1.21 (0.89–1.65)	0.223	1.35 (0.95–1.92)	0.099
	TT	7.6 (5.5–8.3)	1.63 (0.83–3.21)	0.158	1.41 (0.65–3.07)	0.390
	CT+TT	7.6 (5.3–11.9)	1.25 (0.93–1.68)	0.131	1.35 (0.97–1.89)	0.076

1 adj = adjusted for CRP, histological type, weight loss, asbestos exposure, and smoking; CI = confidence interval; HR = hazard ratio; PFS = progression-free survival; Ref. = reference genotype; SNP = single nucleotide polymorphism

### Association of the investigated *HIF1A* SNPs with overall survival

No statistically significant association between polymorphisms and OS was observed in our study, neither in the univariable analysis, nor after adjusting for CRP levels, smoking status, and histological subtype in the multivariable analysis (Table 4), nor when we analyzed patients treated with gemcitabine/cisplatin (Supplementary Table 6) and those treated with pemetrexed/cisplatin (Supplementary Table 7) separately.

**TABLE 4.** Association between investigated polymorphisms and overall survival (N = 234)

SNP	Genotype	OS median (25%–75%)	HR (95% CI)	P	HR_adj_ (95% CI)_adj_	P_adj_
**rs11549465**	CC	18.1 (9.9–28.0)	Ref.		Ref.	
	CT	21.0 (6.8–29.8)	0.85 (0.56–1.30)	0.451	0.75 (0.47–1.19)	0.217
	TT	20.3 (9.1–32.5)	1.00 (0.56–1.77)	0.994	0.85 (0.46–1.59)	0.610
	CT+TT	21.0 (8.7–29.8)	0.89 (0.62–1.28)	0.542	0.78 (0.52–1.16)	0.215
**rsll549467**	GG	18.1 (9.2–28.7)	Ref.		Ref.	
	GA+AA	22.2 (12.6–29.6)	0.90 (0.55–1.48)	0.687	0.95 (0.56–1.60)	0.847
**rs2057482**	CC	19.6 (9.8–27.9)	Ref.		Ref.	
	CT	18.0 (9.8–29.7)	1.07 (0.76–1.50)	0.694	1.07 (0.74–1.52)	0.729
	TT	11.6 (9.4–22.1)	1.44 (0.70–2.95)	0.325	1.43 (0.66–3.10)	0.366
	CT+TT	15.6 (9.8–29.7)	1.11 (0.81–1.53)	0.529	1.10 (0.78–1.55)	0.573

1 adj = adjusted for CRP, histological type, and smoking; CI = confidence interval; HR = hazard ratio; OS = overall survival; Ref. = reference genotype; SNP = single nucleotide polymorphism

## Discussion

This study is the first to investigate the association of *HIF1A* SNPs with response to treatment with cisplatin in combination with pemetrexed or gemcitabine, as well as with PFS and OS in patients with MM. The key finding was a significantly poorer response to chemotherapy in patients carrying the rs11549465 CT genotype compared to CC genotype carriers, after adjusting for weight loss and CRP. No associations were found for rs11549467 or rs2057482 with treatment response in MM. Furthermore, none of the investigated SNPs were associated with PFS or OS.

We are the first to report a statistically significant poorer response to chemotherapy in patients with MM associated with the rs11549465 CT genotype. No other study has investigated the association between *HIF1A* rs11549465 and response to chemotherapy and survival in MM, and there were only a few studies in other cancers. Furthermore, most of these studies were conducted by Chinese researchers and are thus limited to Asian populations.^23^ A study in 741 Chinese patients with histologically confirmed stage III or IV non-small cell lung cancer (NSCLC) reported that carriers of the rs11549465 CC genotype were more likely to respond to platinum-based chemotherapy than those with the TT genotype. They also reported that among NSCLC patients, a higher proportion of rs11549465 TT genotype carriers had higher HIF-1A expression compared to those with the CC genotype, and patients with high HIF-1A expression were significantly more likely to have a poor response to chemotherapy when compared to patients with low expression.^30^ While we observed no association of rs11549465 with OS and PFS in MM patients, among NSCLS patients longer OS and PFS was reported for CC genotype carriers compared to CT or TT genotype carriers.^30^ Another study examining the association between this polymorphisms and response to platinum-based neoadjuvant chemotherapy in locally advanced cervical cancer found a higher likelihood of treatment response in carriers of the CC genotype compared to those with CT or TT genotypes.^31^ Their results are consistent with our finding in MM that the CT genotype is associated with a poorer chemotherapy response. Increased HIF-1A expression and poorer disease prognosis in rs11549465 CT or TT carriers were also reported in a recent study on advanced NSCLC treated with radiotherapy.^32^

A poorer response to chemotherapy in patients carrying the CT genotype could be partially explained by its association with increased expression of HIF-1A. Studies have shown that higher HIF-1A expression activates genes involved in pathways regulating cell proliferation, drug metabolism, apoptosis, autophagy, and angiogenesis, all of which contribute to treatment resistance. The resulting dysregulation may also affect the genes encoding drug transporters such as multidrug resistance protein 1 (MDR1) and multidrug resistance-associated protein 2 (MRP2), the glucose transporter 1 (GLUT-1), the vascular endothelial growth factor (VEGF), and the anti-apoptotic protein Bcl-2.^33,34^ It has a been also shown in isogenic fibrosarcoma cells, that inactivation of HIF-1A can reverse hypoxia-induced cisplatin resistance.^35^ However, this observations do not fully align with our findings, since increased HIF-1A expression was also associated with the rs11549465 TT genotype, not associated with chemotherapy response in our study. Such discrepancies between our results and those of related studies may be due to the complex and diverse mechanisms through which HIF-1A promotes chemo- and radio-resistance in cancer cells.^34^

We found no association between rs11549467 and chemotherapy response, PFS and OS in patients with MM undergoing platinum-based chemotherapy. To our knowledge, no previous studies have investigated this polymorphism in MM patients, while the aforementioned studies in NSCLC and cervical cancer similarly reported no association between rs11549467 and chemotherapy response or survival.^30–32^

In our study, rs2057482 was also not associated with chemotherapy response, PFS or OS in MM patients treated with chemotherapy. To our knowledge, no studies have investigated this polymorphism in MM patients. However, in some other cancers, the findings regarding this polymorphism have been quite contradictory and cannot be directly compared to our results due to different treatment. A study examining the association of *HIF1A* polymorphisms with prognosis in patients with advanced NSCLC treated with radiotherapy found that rs2057482 CT or TT genotypes carriers had shorter PFS and worse OS compared to the CC genotype carriers.^32^ On the other hand, in patients with early-stage (TNM I/II) NSCLC undergoing surgical treatment, the CT and TT genotypes seemed to have a protective effect on OS and DFS compared to the CC genotype. However, when patients of all stages were included in the analysis, no differences were observed between genotypes.^36^ Similarly, in aggressive hepatocellular carcinoma, longer DFS and borderline longer OS following surgical treatment were observed in of the CT or TT genotypes carriers.^37^

These discrepancies suggest that the impact of *HIF1A* polymorphisms on patient survival may be cancer-type or treatment specific. A genotype that is protective in one cancer type could be associated with poorer prognosis in another.^32^ This may be explained by the fact that rs2057482 is located in the 3’ UTR region of the *HIF1A*, near two microRNA binding sites. Studies have shown that polymorphisms in this region can alter microRNA binding sites, thereby affecting mRNA stability. This, in turn, could modify HIF1A expression and consequently influence survival outcomes.^37,38^ Further studies are needed to confirm these hypotheses.

A potential limitation of our study may be, that we observed a deviation of rs11549465 polymorphism from HWE. To verify the accuracy of the KASP genotyping results, we repeated genotyping of this polymorphism in all samples using an independent TaqMan assay and the results were concordant. Despite this, the genotype distribution remained inconsistent with HWE, possibly due to higher frequency of homozygotes for the rare allele (TT). This observed deviation from HWE for rs11549465 may also reflect the nature of our patient cohort, which does not represent a general population as MM is a rare cancer, mostly associated with asbestos exposure. Moreover, studies have shown that *HIF1A* polymorphisms are associated with the risk of developing various cancers.^13,39,40^

The strength of our study lies in the inclusion of as many as 234 patients with MM, which, considering the rarity of this disease, represents a very large cohort and one of the largest genetic association studies in MM both nationally and internationally. Another advantage is that all patients were treated and followed up at the same institution and according to the same protocol. Furthermore, our study included patients from a genetically homogeneous Slovenian population, allowing us to exclude the influence of genetic heterogeneity on our results. On the other hand, this may limit the translation of our findings to other populations. Consequently, we were unable to directly compare our results with those from other studies, which investigated other cancers and were mostly conducted on Asian populations.

Despite being a rare cancer, MM remains a major public health concern due to high mortality and rising incidence among older, asbestos-exposed individuals, unlike younger individuals, in whom incidence is declining.^1,6,20^ Therefore, it is crucial that further studies clarify the mechanisms influencing the course and treatment of MM. To our knowledge, our study is the first worldwide to investigate the role of *HIF1A* polymorphisms in patients with MM. Additionally, it is one of the few studies examining *HIF1A* polymorphisms in cancer within a European population, as most previous studies has been conducted in Asian populations.

Although we found a statistically significant poorer response to chemotherapy in heterozygous patients with the rs11549465 CT genotype after adjustment for weight loss and CRP, we believe that the results of this study do not provide new findings with immediate clinical applicability. Consequently, testing MM patients for these three *HIF1A* polymorphisms has for now, in our opinion, no clinical utility. Nevertheless, our findings may contribute to understanding the role of *HIF1A* polymorphisms in MM and cancer more broadly, but further studies will be necessary to clarify their role. Moreover, since our study included only Slovenian patients, it remains necessary to investigate whether any associations exist between *HIF1A* polymorphisms and chemotherapy response or survival in patients with MM in other populations, particularly non-Asian groups for which data are currently lacking. Further research on *HIF1A* polymorphisms is also needed to more precisely explain their cellular mechanisms and roles in various pathological processes in the body.

## Conclusions

This study is the first to investigate the role of *HIF1A* polymorphisms in MM in the context of platinum-based chemotherapy. Among the variants examined, only the rs11549465 CT genotype was associated with a significantly poorer response to chemotherapy after adjusting for clinical factors. No associations were found between any of the investigated SNPs and OS or PFS. Although our findings do not currently support clinical implementation of *HIF1A* genotyping in MM management, they contribute to the growing body of evidence on the role of hypoxia-related genetic variability in chemotherapy resistance. Further research in larger and more diverse populations is warranted to validate these findings and explore their broader implications in cancer biology and treatment personalization.

## Supplementary Material

Supplementary Material Details
