# Supplementary material for: Genetic variability of HIF1A and response to treatment with cisplatin in combination with pemetrexed or gemcitabine in patients with malignant mesothelioma
Source: Radiol Oncol. 2025 Sep 5;59(3):403–11. doi: 10.2478/raon-2025-0049 (PMC12441883; doi:10.2478/raon-2025-0049)
Supplement: Supplementary file 1 — Supplementary Material Details [file raon-2025-0049_sm.pdf]

Genetic variability of *HIF1A* and response to treatment with cisplatin in combination with pemetrexed or gemcitabine in patients with malignant mesothelioma

Matic Setina, Eva Setina, Ziga Doljak, Katja Goricar, Vita Dolzan, Viljem Kovac

doi: 10.2478/raon-2025-0049

SUPPLEMENTARY TABLE 1. *HIF1A* genotype frequency distribution

Gen	SNP [N]	Genotype	N (%)	MAF	pHWE
HIF1A	rs11549465 [22]	CC	155 (73.1)	17.5	**<0.001**
	p.Pro582Ser	CT	40 (18.9)		
		TT	17 (8.0)		
	rs11549467 [13]	GG	199 (90.0)	5.4	0.077
	p.Ala588Thr	GA	20 (9.0)		
		AA	2 (0.9)		
	rs2057482 [5]	CC	155 (73.1)	18.3	0.311
	c.*45T>C	CT	64 (27.9)		
		TT	10 (4.4)		

HWE = Hardy-Weinberg equilibrium; MAF = minor allele frequency; N = number of patients; [N] = number of patients with missing data for the given parameter; SNP = single nucleotide polymorphism

SUPPLEMENTARY TABLE 2. Association between the investigated *HIF1* polymorphisms and response to gemcitabine/cisplatin therapy (N = 147)

SNP	Genotype	SD/PD N (%)	CR/PR N (%)	OR (95% CI)	P	OR_{adj} (95% CI) $_{adj}$	P_{adj}
**rs11549465**	CC	57 (60.0)	38 (40.0)	Ref.		Ref.	
	CT	16 (76.2)	5 (23.8)	0.47 (0.16–1.39)	0.171	0.52 (0.17–1.64)	0.268
	TT	8 (61.5)	5 (38.5)	0.94 (0.29–3.08)	0.915	1.40 (0.39–5.08)	0.608
	CT+TT	24 (70.6)	10 (29.4)	0.63 (0.27–1.45)	0.275	0.77 (0.32–1.91)	0.578
**rs11549467**	GG	78 (64.5)	43 (35.5)	Ref.		Ref.	
	GA+AA	8 (57.1)	6 (42.9)	1.36 (0.44-4.18)	0.591	1.33 (0.40–4.44)	0.641
**rs2057482**	CC	58 (61.1)	37 (38.9)	Ref.		Ref.	
	CT	27 (60.0)	18 (40.0)	1.05 (0.51–2.16)	0.905	0.77 (0.34–1.75)	0.527
	TT	5 (100.0)	0 (0.0)	/	0.154*	/	/
	CT+TT	32 (64.0)	1 (36.0)	0.88 (0.43–1.79)	0.728	0.63 (0.28–1.41)	0.263

adj = adjusted for weight loss and ECOG performance status; CR = complete response; CI = confidence interval; N = number of patients; PD = progressive disease; PR = partial response; Ref. = reference genotype; OR = odds ratio; SD = stable disease; SNP = single nucleotide polymorphism; * = calculated using Fisher's exact test

SUPPLEMENTARY TABLE 3. Association between the investigated *HIF1* polymorphisms and response to pemetrexed/cisplatin therapy (N = 79)

SNP	Genotype	SD/PD N (%)	CR/PR N (%)	OR (95% CI)	P	OR_{adj} $(95\% CI)_{adj}$	P_{adj}
**rs11549465**	CC	35 (66.0)	18 (34.0)	Ref.		Ref.	
	CT	15 (78.9)	4 (21.1)	0.52 (0.15–1.79)	0.300	0.53 (0.15–1.86)	0.318
	TT	2 (66.7)	1 (33.3)	0.97 (0.08–11.46)	0.982	1.10 (0.09–13.59)	0.940
	CT+TT	17 (77.3)	5 (22.7)	0.57 (0.18–1.80)	0.340	0.59 (0.18–1.90)	0.374
**rs11549467**	GG	48 (67.6)	23 (32.4)	Ref.		Ref.	
	GA+AA	6 (85.7)	1 (14.3)	0.35 (0.04–3.06)	0.341	0.36 (0.04–3.20)	0.358
**rs2057482**	CC	37 (67.3)	18 (32.7)	Ref.		Ref.	
	CT	14 (82.4)	3 (17.6)	0.44 (0.11–1.73)	0.240	0.45 (0.11–1.78)	0.252
	TT	2 (40.0)	3 (60.0)	3.08 (0.47–20.12)	0.239	3.51 (0.51–24.29)	0.203
	CT+TT	16 (72.7)	6 (27.3)	0.77 (0.26–2.30)	0.641	0.79 (0.26–2.42)	0.685

adj = adjusted for pain; CR = complete response; CI = confidence interval; N = number of patients; PD = progressive disease; PR = partial response; Ref. = reference genotype; OR = odds ratio; SD = stable disease; SNP = single nucleotide polymorphism

SUPPLEMENTARY TABLE 4. Association between the investigated *HIF1* polymorphisms and progression-free survival in patients treated with gemcitabine/cisplatin therapy (N = 149)

SNP	Genotype	PFS median (25%–75%)	HR (95% CI)	P	HR_{adj} $(95\% CI)_{adj}$	P_{adj}
**rs11549465**	CC	8.6 (5.7–13.0)	Ref.		Ref.	
	CT	7.9 (5.1–11.7)	1.05 (0.64–1.73)	0.843	1.07 (0.64–1.82)	0.788
	TT	6.6 (3.9–18.7)	1.00 (0.56–1.80)	0.996	0.97 (0.50–1.88)	0.937
	CT+TT	7.9 (4.8–13.1)	1.03 (0.69–1.54)	0.888	1.04 (0.66–1.61)	0.878
**rs11549467**	GG	7.9 (5.3–13.0)	Ref.		Ref.	
	GA+AA	10.5 (7.7–16.0)	0.78 (0.44–1.38)	0.393	0.83 (0.46–1.48)	0.522
**rs2057482**	CC	9.1 (5.6–13.9)	Ref.		Ref.	
	CT	7.9 (5.3–11.9)	1.20 (0.83–1.74)	0.322	1.41 (0.94–2.12)	0.092
	TT	7.8 (7.6–22.3)	1.14 (0.42–3.11)	0.800	1.31 (0.47–3.67)	0.603
	CT+TT	7.8 (5.3–11.9)	1.20 (0.84–1.71)	0.320	1.40 (0.95–2.07)	0.087

adj = adjusted for CRP, histological type, weight loss, asbestos exposure, and smoking; CI = confidence interval; PFS = progression-free survival; Ref. = reference genotype; HR = hazard ratio; SNP = single nucleotide polymorphism

SUPPLEMENTARY TABLE 5. Association between the investigated *HIF1* polymorphisms and progression-free survival in patients treated with pemetrexed/cisplatin therapy (N = 82)

SNP	Genotype	PFS median (25%–75%)	HR (95% CI)	P	HR_{adj} (95% CI) $_{adj}$	P_{adj}
**rs11549465**	CC	8.6 (5.7–13.0)	Ref.		Ref.	
	CT	7.9 (5.1–11.7)	1.05 (0.64–1.73)	0.843	1.07 (0.64–1.82)	0.788
	TT	6.6 (3.9–18.7)	1.00 (0.56–1.80)	0.996	0.97 (0.50–1.88)	0.937
	CT+TT	7.9 (4.8–13.1)	1.03 (0.69–1.54)	0.888	1.04 (0.66–1.61)	0.878
**rs11549467**	GG	7.9 (5.3–13.0)	Ref.		Ref.	
	GA+AA	10.5 (7.7–16.0)	0.78 (0.44–1.38)	0.393	0.83 (0.46–1.48)	0.522
**rs2057482**	CC	9. (5.6–13.9)	Ref.		Ref.	
	CT	7.9 (5.3–11.9)	1.20 (0.83–1.74)	0.322	1.41 (0.94–2.12)	0.092
	TT	7.8 (7.6–22.3)	1.14 (0.42–3.11)	0.800	1.31 (0.47–3.67)	0.603
	CT+TT	7.8 (5.3–11.9)	1.20 (0.84–1.71)	0.320	1.40 (0.95–2.07)	0.087

adj = adjusted for CRP and histological type; CI = confidence interval; PFS = progression-free survival; Ref. = reference genotype; HR = hazard ratio; SNP = single nucleotide polymorphism

SUPPLEMENTARY TABLE 6. Association between the investigated *HIF1* polymorphisms and overall survival in patients treated with gemcitabine/cisplatin therapy (N = 152)

SNP	Genotype	OS median (25%–75%)	HR (95% CI)	P	HR_{adj} (95% CI) $_{adj}$	P_{adj}
**rs11549465**	CC	18.2 (10.1–26.7)	Ref.		Ref.	
	CT	12.8 (6.6–47.8)	0.84 (0.47–1.50)	0.558	0.79 (0.43–1.44)	0.437
	TT	20.3 (9.1–50.8)	0.91 (0.46–1.83)	0.796	0.73 (0.35–1.52)	0.394
	CT+TT	18.0 (6.9–47.8)	0.87 (0.54–1.39)	0.557	0.76 (0.47–1.25)	0.280
**rs11549467**	GG	18.0 (8.7–26.7)	Ref.		Ref.	
	GA+AA	22.7 (10.5–29.6)	0.90 (0.49–1.64)	0.723	0.98 (0.53–1.82)	0.959
**rs2057482**	CC	19.6 (9.1–26.5)	Ref.		Ref.	
	CT	18.1 (11.2–29.7)	0.95 (0.64–1.43)	0.815	0.97 (0.64–1.48)	0.901
	TT	22.1 (11.6–*)	1.00 (0.32–3.20)	0.994	1.20 (0.37–3.84)	0.760
	CT+TT	18.1 (11.2–29.7)	0.96 (0.65–1.42)	0.826	0.99 (0.66–1.48)	0.960

adj = adjusted for CRP, histological type, asbestos exposure, and smoking; CI = confidence interval; OS = overall survival; Ref. = reference genotype; HR = hazard ratio; SNP = single nucleotide polymorphism; * = not reached

SUPPLEMENTARY TABLE 7. Association between the investigated *HIF1* polymorphisms and overall survival in patients treated with pemetrexed/cisplatin therapy (N = 82)

SNP	Genotype	OS median (25%–75%)	HR (95% CI)	P	HR_{adj} (95% CI) $_{adj}$	P_{adj}
**rs11549465**	CC	15.4 (9.4–29.7)	Ref.		Ref.	
	CT	21.8 (13.1–28.4)	0.88 (0.47–1.67)	0.700	0.77 (0.37–1.62)	0.493
	TT	12.1 (6.4–23.6)	1.17 (0.42–3.31)	0.762	0.94 (0.28–3.10)	0.916
	CT+TT	21.8 (12.1–28.4)	0.94 (0.53–1.67)	0.825	0.81 (0.41–1.57)	0.528
**rs11549467**	GG	18.3 (9.4–29.7)	Ref.		Ref.	
	GA+AA	16.7 (12.6–29.8)	0.93 (0.40–2.18)	0.869	0.78 (0.28–2.21)	0.639
**rs2057482**	CC	21.0 (10.0–28.7)	Ref.		Ref.	
	CT	15.0 (9.5–29.8)	1.38 (0.75–2.54)	0.304	0.88 (0.39–1.97)	0.756
	TT	9.9 (9.4–12.1)	1.98 (0.77–5.12)	0.157	1.48 (0.52–4.27)	0.463
	CT+TT	14.9 (9.4–29.8)	1.49 (0.86–2.60)	0.159	1.02 (0.51–2.05)	0.945

adj = adjusted for CRP; CI = confidence interval; OS = overall survival; Ref. = reference genotype; HR = hazard ratio; SNP = single nucleotide polymorphism
